# Water-Use Efficiency and Physiological Responses of Juvenile Northern River Shrimp (*Cryphiops caementarius*) Cultured in Biofloc Systems Using Molasses and Chancaca as Carbon Sources

**DOI:** 10.3390/ani16030470

**Published:** 2026-02-03

**Authors:** Carlos Andres Mendez, David Ulloa Walker, Camila Salvador, Carla Galleguillos, María Cristina Morales

**Affiliations:** 1 Programa Doctorado en Acuicultura, Facultad de Ciencias del Mar, Universidad Católica del Norte, Coquimbo 1781421, Chile; 2 Departamento de Acuicultura, Facultad de Ciencias del Mar, Universidad Católica del Norte, Larrondo 1281, Coquimbo 1781421, Chile; camila.salvador@ucn.cl (C.S.); carla.galleguillos@ucn.cl (C.G.); mcmorale@ucn.cl (M.C.M.); 3 Laboratorio de Fisiología y Genética Marina (FIGEMA), Departamento de Acuicultura, Facultad de Ciencias del Mar, Universidad Católica del Norte, Coquimbo 1781421, Chile; 4 Imenco Aqua Chile, Ruta 5 Sur, Km 1025. Modulo-15A., Puerto Mont 5480000, Chile

**Keywords:** biofloc technology (BFT), Hsp70, aquaculture sustainability, freshwater shrimp, water quality

## Abstract

Biofloc technology (BFT) is based on the reutilization of nitrogenous waste generated by cultured organisms through the biotransformation of these compounds primarily into microbial biomass, allowing a reduction in water exchange. The aim of this study was to evaluate BFT as a water-saving culture strategy, using two carbon sources (chancaca and molasses), and to assess its effects on water-use efficiency, growth performance, digestive enzyme activity, and physiological responses in juvenile northern river shrimp (*Cryphiops caementarius*). The experiment was conducted in triplicate using 400 L fiberglass tanks, with an initial stocking density of 75 shrimp m^−2^ and an average individual weight of 0.85 ± 0.65 g, over a 157-day rearing period. Water quality parameters were maintained within suitable ranges throughout the study. Significant differences were observed in the composition of bacterial and plankton communities among the biofloc treatments, whereas no significant differences were detected in growth performance or digestive enzyme activities. Heat shock protein 70 (Hsp70), a stress-related biomarker indicative of physiological responses, exhibited higher levels in the biofloc treatment supplemented with molasses. Overall, BFT treatments reduced water exchange by 81.6% while maintaining comparable biological performance to the control, indicating that biofloc technology represents a water-efficient and environmentally sustainable culture approach for juvenile *Cryphiops caementarius*, an endemic freshwater shrimp species, particularly in water-limited regions of northern Chile.

## Simple Summary

Shrimp farming is an important source of food and income, but it often requires large amounts of water and can generate waste that negatively affects rivers and lakes. This study explored biofloc technology as a water-saving strategy for rearing juvenile northern river shrimp (*Cryphiops caementarius*), an endemic freshwater species from Chile that is currently classified as vulnerable. Biofloc systems improve water quality by recycling waste nutrients into microbial aggregates that remain suspended in the water. Two natural sugar-based carbon sources, molasses and chancaca, were used to support biofloc development. Over a 157-day experimental period, shrimp reared in biofloc systems were compared with shrimp maintained under clear-water conditions. Shrimp growth performance was similar among all systems; however, biofloc treatments reduced water exchange by 81.6%. The type of carbon source influenced the structure of the microbial community, and molasses was associated with changes in the stress-related biomarker heat shock protein 70 (Hsp70), linked to physiological responses. These results indicate that biofloc technology can markedly reduce water use while maintaining biological performance, offering a practical and environmentally sustainable approach for the culture of an endemic freshwater shrimp and contributing to the conservation of Chile’s river ecosystems.

## 1. Introduction

Aquaculture, as an activity aimed at the cultivation and controlled production of hydrobiological organisms, has grown, surpassing capture fisheries as the main producer of aquatic animals. Global aquaculture production reached an unprecedented figure of 130.9 million tons, of which 94.4 million tons correspond to aquatic animals, representing 51% of the total aquatic animal production [1]. The productive growth of aquaculture is directly related to population demographic growth, due to the increased demand for food, such as protein sources, and the stagnation of fisheries. However, this accelerated growth has impacts on the environment, and some of the most questioned are the use and/or consumption of large volumes of water, the generation of effluents, habitat alteration, escapes of cultured species, and the consequent interaction with native species [2,3,4,5,6,7,8]. Among farming systems that operate with zero or minimal water exchange, biofloc technology (BFT) has gained attention due to its capacity to reduce nutrient-rich effluent discharge, minimize risks associated with species escape and disease transmission, and improve water-use efficiency through limited water renewal [9,10,11,12]. In shrimp culture, BFT has been reported to maintain growth performance while modulating microbial communities and physiological responses, and providing additional nutritional contributions through microbial biomass, depending on system configuration and management practices [13,14,15,16]. BFT has been successfully applied in freshwater fish such as tilapia (*Oreochromis aureus*, *O. niloticus*, *O. mossambicus*) [17,18,19]; carp (*Cyprinus carpio*) [20,21]; tambaqui (*Colossoma macropomum*) [22,23]; and in marine crustaceans such as *Penaeus vannamei* [9,11,24], *Litopenaeus stylirostris* [25], *Farfantepenaeus paulensis* [26], *Fenneropenaeus merguiensis* [27], *P. monodon* [28,29,30], as well as in freshwater prawn *Macrobrachium rosenbergii* [12,31,32,33,34], *Cherax cainii* [35], *Astacus leptodactylus* [36], and *Procambarus clarkii* [37,38].

BFT is based on the assimilation of inorganic nitrogen by the microbial community present in the culture medium [17,39]. The proportion and predominance of some of these microbial groups are determined by the interaction of various biotic and abiotic factors, presenting an ecological succession in the formation and development of the biofloc over time [40,41,42]. One aspect used to differentiate BFT-based systems is the coloration of the culture medium, which serves as a first practical field tool, although it must be validated with the analysis of physicochemical water quality parameters [43]. In those systems where microalgae predominate, they are known as green water systems, with photoautotrophic characteristics. In contrast, those where the coloration fluctuates from brownish and dark brown tones show a predominance of heterotrophic bacteria over nitrifying bacteria and microalgae [44,45]. In biofloc-based systems, heterotrophic bacteria use organic carbon as their primary energy source and assimilate inorganic nitrogen, particularly ammonia, for the synthesis of cellular proteins, thereby promoting rapid microbial growth and biomass production [46]. This process contributes to mitigating the accumulation of potentially toxic nitrogenous compounds and enhances nutrient recycling by converting waste-derived nitrogen into microbial protein, which may enrich the nutritional environment of the culture system [47]. To stimulate this metabolic pathway, the controlled addition of an external carbon source is required to adjust the carbon-to-nitrogen (C:N) ratio, which is commonly maintained within ranges that favor heterotrophic dominance over autotrophic nitrification [48]. A wide variety of organic carbon substrates have been applied for this purpose and can be classified, according to their carbon release kinetics, as slow- or fast-release sources [49,50,51,52,53]. This release dynamic plays a key role in carbon availability and bacterial activity: slow-release substrates tend to provide a more stable carbon supply, promoting gradual ammonia assimilation and greater system stability, whereas fast-release sources may induce abrupt bacterial proliferation, increasing biofloc formation and leading to greater fluctuations in water quality [54,55]. Among the most widely used carbon sources in different studies related to BFT are molasses, glycerol, wheat flour, starch, among others [56,57,58,59]. It has been described that the application of different external carbon sources influences water quality in culture systems, as well as animal performance and the quality and composition of floc [60,61,62,63,64,65,66,67,68,69].

Considering the importance of carbon source selection for biofloc development and overall system performance, this study evaluated the use of two fast-release carbon sources: molasses, a by-product of sugar production widely applied in biofloc systems, and chancaca, a locally available sugar concentrate traditionally produced and commercialized in Chile. The primary objective of this study was to evaluate biofloc technology as a water-saving culture strategy using different carbon sources, and to assess its effects on water-use efficiency, growth performance, digestive enzyme activity, and stress-related physiological responses in the northern river shrimp *Cryphiops caementarius*. This species is an endemic freshwater crustacean from Chile and is currently classified as vulnerable according to the official national conservation assessment (Ministerio del Medio Ambiente, Chile, https://clasificacionespecies.mma.gob.cl/) (accessed on 12 July 2025) indicating an elevated risk of population decline under natural conditions. In addition, *C. caementarius* holds considerable ecological and socio-economic importance in its native range. Despite this relevance, aquaculture research on *C. caementarius* remains limited, particularly with respect to intensive and water-efficient production systems [50]. Consequently, the evaluation of alternative culture strategies, such as biofloc technology, is especially relevant for this species, as it may contribute to reducing water consumption while maintaining biological performance under controlled conditions.

## 2. Materials and Methods

The research was performed in the Universidad Católica del Norte (UCN), Guayacán campus, in the city of Coquimbo, Chile, located at coordinates 29°57′56.30″ South and 71°21′11.00″ West, specifically in the Mass Crustacean Culture Laboratory (CMC) of the Faculty of Marine Sciences, Department of Aquaculture. All animal procedures in this study were conducted according to the guidelines for the care and use of the Mass Crustacean Culture Laboratory. Animal studies were reviewed and approved by the Ethics and Biotechnology Committee of the Universidad Católica del Norte (UCN), Coquimbo, Chile (CEC UCN No. 45; Approval Date: 16 November 2021).

### 2.1. Experimental Design and Conditions

For the initial formation of the biofloc, a 500 L tank was prepared with previously analyzed, filtered, and disinfected freshwater (treated with 15 ppm chlorine and neutralized after 24 h with 5 mg L^−1^ sodium thiosulfate), and maintained under continuous aeration. Extruded feed was added as the primary organic carbon source, while urea (46%) was supplied as a nitrogen source to promote microalgae proliferation. Sodium bicarbonate was used to provide alkalinity, and the system was inoculated with microalgae—mainly Chlorophyceae—along with the commercial bacterial product EcoPro (EcoMicrobials LLC., Miami, FL, USA), which contains stable spores of *Paenibacillus polymyxa*, *Bacillus megaterium*, *Bacillus licheniformis*, and *Bacillus subtilis* (minimum guaranteed concentration of 5.5 × 10^8^ CFU g^−1^). Once the biofloc reached a stable condition, it was divided into two 500 L tanks, each receiving one of the carbon sources evaluated in this study: molasses or chancaca. The carbon sources were diluted in tank water and applied evenly to each treatment to maintain a carbon-to-nitrogen (C:N) ratio of 12:1, following the guidelines proposed by Avnimelech [17] and Ebeling et al. [70]. The required carbon input was calculated based on the target C:N ratio and adjusted according to the estimated carbon content of each source, considering molasses (approximately 30% carbon) and chancaca (approximately 36% carbon) as fast-release substrates. Carbon sources were added daily in dissolved form and in small, incremental doses to minimize abrupt changes in water quality. Total ammonia nitrogen (TAN) concentrations were monitored throughout the experimental period and used as an indirect indicator to fine-tune carbon additions and maintain system stability. The experimental BFT system for each carbon source consisted of three 400 L rectangular units (1.46 m^2^ each), independently connected to a 500 L reserve tank and a 200 L sedimentation tank. Water circulation was ensured by a submersible pump (Atman AT-105, Guangzhou Ample Technology Co., Ltd., Zhongshan, China, Qmax. 3000 L h^−1^) to preserve the planktonic community and maintain water quality [9,71]. In contrast, the control treatment (clear water) had no recirculation and received a 50% weekly water exchange. The entire system operated under natural photoperiod, with a constant temperature of 23 °C maintained by 200 W submersible heaters (Whale VK-1000 Zhongshan Enjoyroyal Appliance Co., Ltd., Zhongshan, China). Aeration was provided by microperforated hoses connected to a 2.5 HP blower (Sweetwater, Pentair, Golden Valley, MN, USA). All tanks contained 12 PVC shelters (10–15 cm long, 30–40 mm diameter, joined in groups of 3–4). In the BFT treatments, water addition was limited to compensating for evaporation and solids removal. The different water exchange regimes applied to the control and biofloc treatments were inherent to the operational principles of each culture system. The control treatment followed a conventional management strategy with periodic water renewal, whereas the biofloc treatments were operated under minimal water exchange conditions, consistent with standard BFT practices.

### 2.2. River Shrimp Stock

A total of 990 juveniles of *C. caementarius* were used, produced in the UCN laboratory of CMC from ovigerous wild females captured from the natural environment (Limarí River, IV Region, Coquimbo). The organisms were acclimatized to the rearing temperature for three weeks before starting the trial. Each of the rearing tanks was stocked with 110 juveniles with an average body weight of 0.85 ± 0.65 g, with an initial density of 75 shrimp m^−2^. The animals were fed daily with formulated trout feed at 5% of their biomass. The nutritional composition of the diet was 48.5% crude protein, 18.5% lipids, 1.9% fiber, 12% ash, and 10% moisture. A single pellet size of 3 mm was used throughout the experimental period.

### 2.3. Water Quality Parameters

Daily during the experiment, dissolved oxygen (DO), temperature, and pH were measured using an HQ40d multiparameter instrument (Hach Company, Loveland, CO, USA). Once a week, total ammonia as nitrogen (TAN), nitrate as nitrogen, nitrite as nitrogen, total suspended solids (TSS), total suspended volatiles (VSS), floc volume (FV), total alkalinity, and hardness were measured. The determination of nitrogen compounds was carried out using the HACH company protocol. The salicylate method was used for Total Ammonia Nitrogen (TAN), the cadmium reduction method for nitrate, and the diazotization method for nitrite. Measurements were performed using a DR 3900 Spectrophotometer (HACH, Loveland, CO, USA). TSS and VSS were measured using 2540D and 2540E method described in American Public Health Association [72]. FV was measured on Imhoff cones (1000-0010 Vitlab, Grossostheim, Germany) following the methodology proposed by Avnimelech and Kochba [73]. Total alkalinity was determined by the Bromophenol blue titration method, with the HI3811alkalinity kit (Hanna Instruments, Smithfield, RI, USA), and total harness was determined by the Titration Method with EDTA 5-B (Hach Company, Loveland, CO, USA).

### 2.4. Quantification of Total Culturable Bacteria and Vibrio spp.

Biofloc samples were centrifuged at 4200× *g* for 10 min, after which excess supernatant was removed. The resulting pellet was weighed using an analytical balance and resuspended in 5 mL of sterile saline solution (0.85% NaCl) prior to plating. Both control and biofloc samples were inoculated onto Petri dishes containing Plate Count Agar (PCA) and thiosulfate–citrate–bile salts–sucrose (TCBS) agar using the surface spread technique to quantify total cultivable bacteria (TCB) and *Vibrio* spp., respectively. PCA plates were incubated at 18 °C for 5 days, whereas TCBS plates were incubated at 18 °C for up to 48 h. Developed colonies were manually counted, and bacterial abundance was expressed as colony-forming units per milliliter (CFU mL^−1^) [74]. Negative controls consisting of sterile TCBS agar plates were included in each microbiological assay, and no bacterial growth was observed, confirming the absence of cross-contamination during sample processing. A limitation of this study is that bacteriological analysis was restricted to culturable bacteria, which represent only a small fraction of the microbial community in complex environments such as biofloc systems.

### 2.5. Determination of Phytoplanktonic and Zooplanktonic Communities

At the end of the experiment, 1 L of water was collected from each culture tank, filtered, and the concentrated material preserved in 50 mL bottles with 5% formalin, following the protocol of Azim [75]. Plankton quantification was conducted using a Sedgwick–Rafter counting chamber under a light microscope (Carl Zeiss, Primo Star, Oberkochen, Germany; 10×–40×). Identification of microalgae and planktonic groups was performed using photographs obtained with a Canon EOS Rebel T5 camera (Canon, Tokyo, Japan) and the corresponding taxonomic keys [76,77]. Microalgal density was expressed as cells mL^−1^. To characterize other organisms (ciliates, rotifers, cladocerans, and nematodes), five 5 mL subsamples from each tank were fixed with 5% formalin and examined directly in the laboratory. Zooplankton were identified to the generic level and quantified as individuals mL^−1^ [78,79,80].

### 2.6. Measurement of Growth Performance Parameters

Weight measurements were taken using a Mettler PJ3600 digital scale (Mettler, Columbus, OH, USA) with a precision of 0.01 g. At the end of the experiment, the following performance parameters were evaluated: specific growth rate (SGR)  = (lnTW_2_ − lnTW_1_) × 100/(T_2_ − T_1_); where TW_1_ and TW_2_ are total weight at days T_1_ (start of the experiment) and T_2_ (after 157 days); survival rate (SR%) = final shrimp number/initial shrimp number) × 100; feed conversion ratio (FCR) = offered feed (g)/(final biomass (g) − initial biomass (g)); productivity (g m^−2^) = final biomass (g)/Area of the experimental unit (m^2^).

### 2.7. Sample Collection

Digestive enzyme activity and physiological responses in shrimp were measured at the end of the experimental period. Twelve shrimp per treatment were removed from the tanks and placed on ice at low temperature (<10 °C) to induce anesthesia prior to sampling. Once sacrificed, each shrimp was dissected into two segments: cephalothorax and abdomen. The cephalothorax was used for the analysis of digestive enzyme activity, while the abdomen was used for physiological response assays. Both segments were placed in sterile 2 mL Eppendorf tubes with RNA Later™ solution (Invitrogen, Thermo Fisher Scientific corporation, Barcelona, Spain) and stored at −80 °C until further analysis.

#### 2.7.1. Digestive Enzyme Analysis

For amylase enzyme activity, Bernfeld’s method [81] was used. A tube with 500 µL of substrate (1% potato starch dissolved in 0.02 M phosphate buffer at pH 6.9 with 0.006 M NaCl) was incubated, and the reaction was initiated by adding 100 µL of enzyme extract, incubating the mixture for 5 min at 25 °C. After the incubation period, 1 mL of the reactive solution (1 g of dinitrosalicylic acid dissolved in 50 mL of distilled water with 30 g of sodium-potassium tartrate tetrahydrate, plus 20 mL of 2 N NaOH, brought to a final volume of 100 mL) was added. To stop the reaction, the mixture was incubated in boiling water for 5 min. It was then cooled for 10 min at room temperature by adding 10 mL of distilled water. A 3 mL aliquot was taken, and absorbance was recorded at 540 nm. Using a standard maltose curve treated in the same way as the samples, a curve was constructed to determine absorbance at various concentrations. Trypsin was performed by the specific methods of Worthington [82]. The trypsin substrate p-Toluene-sulfonyl-l-arginine methyl ester (TAME) was used as the substrate. To 300 µL of TAME, 100 µL of enzyme extract, and 2600 µL of tris-HCl buffer at pH 8.1 were added to the mixture. Absorbance was measured at 247 nm and 25 °C every min for the first 6 min of incubation. Protein concentrations were determined in the enzyme extracts by the method of Lowry et al. [83] with bovine albumin as the standard to establish the specific activities of the enzymes.

#### 2.7.2. Physiological Responses—Heat Shock Protein 70 (Hsp70)

The quantification of Hsp70 was performed using indirect ELISA, with monospecific polyclonal anti-epitope antibodies for Hsp70, developed in mice immunized with the synthetic epitope peptide [84,85]. The ELISA plates were coated with 40 ng µL^−1^ of total proteins, in a volume of 50 µL in carbonate-bicarbonate buffer (pH 9.6). The plates were then blocked with 5% skim milk dissolved in phosphate-buffered saline (PBS) at 37 °C for 2 h. The plates were washed three times with PBS, and the first antibody, mouse IgG (1:400 dilution) against the invertebrate Hsp70 epitope, was applied and incubated overnight at 4 °C. The plates were washed three times with 1% PBS. The second antibody used was goat Immunoglobulin G (IgG) at 1:2500 dilution, and anti-mouse IgG-HRP (horseradish peroxidase conjugate), which was incubated for 60 min at room temperature. The plates were then washed and developed with the Tetramethylbenzidine (TMB) substrate for 20 min at room temperature in the dark. The reaction was stopped with sulfuric acid, and the absorbance was read at 450 nm using a spectrophotometer [86]. The quantification of Hsp70 was expressed in absorbance values (nm).

### 2.8. Statistical Analysis

Data analysis was performed using IBM^®^ SPSS^®^ Statistics 20.0 software and Microsoft Office Professional Plus 2013. All data were expressed as mean ± standard deviation (SD). The data was tested for normality and heterogeneity of variance using Kolmogorov–Smirnov test and Levene’s, respectively. The independent sample *t* test was utilized in testing the significance of differences between the two as carbon source (molasses and chancaca). One-way ANOVA was applied to the water quality parameters. Growth performance, digestive enzyme, and Hsp 70 levels were compared among treatments using the non-parametric Kruskal–Wallis test to detect significant differences among groups. Culture tanks were considered the experimental units (n = 3 per treatment), whereas individual shrimp sampled within each tank were treated as subsamples. For all growth, enzymatic, and physiological variables, individual measurements were averaged at the tank level prior to statistical analysis. Consequently, all statistical comparisons among treatments were performed using tank means, thereby avoiding pseudoreplication and ensuring independence of observations. Differences were considered statistically significant at *p* < 0.05 [87].

## 3. Results

### 3.1. Water Quality 

The physical and chemical parameters of water quality monitored throughout the experiment are presented in Table 1. The mean values of temperature (<25 °C), dissolved oxygen (>8 mg L^−1^), and pH (>7.7) were similar across all treatments, with no present statistically significant differences (*p* > 0.05). Regarding the nitrogenous compounds, significant differences were observed only in NO_3_ (*p* < 0.05). The nitrate, total hardness, and TSS values were lower in the control. The total alkalinity value was higher in the control than in the BFT systems.

### 3.2. Total Microorganism Present

The total cultivable bacteria counts are presented in Table 2. The total bacteria count (1.12 and 4.97 × 10^4^ CFU mL^−1^) was significantly (*p* < 0.05) higher in the molasses than in the chancaca. The *Vibrio* spp. was only found in the molasses systems, with a concentration of 4.28 ± 1.51 × 10^3^ CFU mL^−1^. Overall, the concentrations of phytoplankton and zooplankton in the BFT were similar; all systems contained unicellular algae, bacterial communities, protozoa, rotifers, etc. However, the number of organisms present varied between the two culture systems. In chancaca, a lower number of microalgae and a higher number of zooplankton were found compared to molasses. Zooplankton community presented 12 genera from six different groups (Table 3).

### 3.3. Growth Performance

The growth performance results are summarized in Table 4. The final weight, Survival, feed conversion ratio, specific growth rate, and productivity of shrimp did not present significant differences among the treatments (*p* > 0.05) during all 157 experimental days.

### 3.4. Digestive Enzymes

The results of specific enzymatic activity for trypsin and amylase are presented in Table 5. No significant differences (*p* > 0.05) were observed among the treatments.

### 3.5. Heat Shock Protein 70 (Hsp70) Response

**Figure 1:**
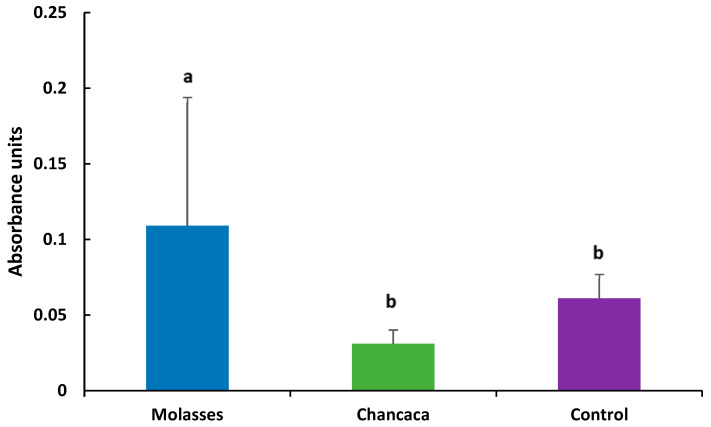
Detection of Hsp70 by indirect ELISA, expressed as mean absorbance units (450 nm) ± standard deviation (SD) per treatment (n = 12). Statistical differences among treatments were evaluated using the Kruskal–Wallis test. Different letters (a, b) indicate significant differences among treatments (*p* < 0.05).

The Hsp70 response is shown in Figure 1. The values of Hsp70 were significantly higher in molasses (0.109 ± 0.085) compared to chancaca (0.031 ± 0.009) and the control (0.061 ± 0.056) (*p* < 0.05).

## 4. Discussion

Water quality parameters are critical in aquaculture, as deviations from optimal ranges can negatively affect productivity and lead to economic losses [88]. In BFT systems, these parameters may vary depending on the carbon source used [89,90]. In this study, all measured variables remained within the recommended ranges for the species and for proper biofloc functioning [91,92,93,94]. Among nitrogenous compounds, TAN and nitrite are the most relevant due to their toxicity [95,96]. Both remained within safe limits, with TAN ≤ 1 mg L^−1^ considered acceptable for fish and crustacean farming [89], and nitrite maintained below the thresholds suggested for BFT systems (<2 mg L^−1^ [32] and ideally <1 mg L^−1^ [89]). Although nitrate tends to accumulate in BFT and becomes critical above 400 mg L^−1^ NO_3_-N [97,98,99], levels in this study remained non-critical. Overall, both carbon sources effectively supported the control of nitrogen compounds by promoting heterotrophic and nitrifying bacteria or microalgae, thereby converting ammonia into microbial protein, nitrate, or algal biomass [17,70].

In this study, no significant differences were observed in FV and TSS (*p* > 0.05) among the biofloc treatments, and the values remained within the recommended range for penaeid species [100,101,102,103]. Alkalinity plays an important role in the formation of biofloc by heterotrophic bacteria, in the nitrification process of chemoautotrophic bacteria, and in pH control [70,104]. Therefore, recommended values should range between 100 and 150 mg CaCO_3_ L^−1^ [48]. In general, in BFT systems, alkalinity and pH levels tend to decrease, while nitrogenous compounds tend to increase [105]. In the case of *M. rosenbergii*, recommended alkalinity values exceed 120 mg L^−1^ as CaCO_3_ [106]. For *C. caementarius*, Mendez et al. [107] previously reported experimental alkalinity levels ranging from 208.8 to 255.0 mg CaCO_3_ L^−1^. In aquaculture, a moderate degree of water hardness, generally between 25 and 100 mg L^−1^, is considered beneficial for freshwater crustaceans. In juvenile *M. rosenbergii*, hardness levels of up to 1000 mg CaCO_3_ L^−1^ have been reported to be tolerated without negative effects on survival or growth, highlighting the physiological relevance of mineral-rich waters. Calcium and magnesium play essential roles in freshwater crustaceans, including osmoregulation, exoskeleton mineralization, and molting processes [108]. However, optimal hardness and alkalinity ranges are species-specific and strongly influenced by the natural hydrochemical conditions of the organism’s habitat [109]. According to reports from the Peruvian Marine Institute [110], river systems inhabited by *C. caementarius* exhibit water hardness values ranging from 154 to 564 mg CaCO_3_ L^−1^. Although the hardness levels recorded in the present study were higher than those typically reported, they reflect the mineral-rich freshwater conditions characteristic of river basins in northern Chile. Considering the ecological distribution and physiological adaptation of *C. caementarius* to hard-water environments, the alkalinity and hardness levels observed in this study can be regarded as environmentally realistic and physiologically relevant for this species. The absence of adverse effects on survival or growth further suggests that these conditions did not impose a significant physiological constraint under the experimental conditions evaluated. The difference in SSV could be due to the composition of the carbon source, as it has been described that molasses contains higher levels of potassium, iron, and manganese compared to other carbon sources such as starch [111,112]. Furthermore, it could be related to the bacterial biomass in the cultivation system, as SSV is considered a measure of bacterial biomass [113].

Bioflocs are complex aggregates composed of bacteria, fungi, algae, protozoa, zooplankton, and particulate organic matter embedded in an extracellular matrix [114,115,116]. Both heterotrophic and autotrophic bacteria play a key role in nutrient recycling and water quality control within BFT systems [54,117]. In biofloc-based culture of Macrobrachium rosenbergii, total bacterial abundances ranging from 0.88 to 4.32 × 10^8^ CFU mL^−1^ have been reported using different carbon sources, with heterotrophic bacteria levels between 6.33 and 383.33 × 10^8^ CFU mL^−1^ [57,118]. Similarly, Burford et al. [119] reported bacterial concentrations of 3–5 × 10^7^ CFU mL^−1^ in Penaeus vannamei culture, while Khanjani et al. [120] documented heterotrophic bacterial levels of approximately 3.4 × 10^7^ CFU mL^−1^. The bacterial abundances observed in the present study were comparatively lower, which may be attributed to two main factors. First, the carbon-to-nitrogen (C:N) ratio applied (12:1) is known to favor the development of chemoautotrophic nitrifying bacteria rather than heterotrophic populations typically promoted at higher C:N ratios (15–20:1) [121,122]. Second, salinity is a critical driver of heterotrophic bacterial proliferation and nitrification dynamics in BFT systems [118,123]; therefore, comparisons with marine or brackish-water shrimp species should be interpreted cautiously, given the freshwater conditions and species-specific responses of *C. caementarius*. In BFT systems, *Vibrio* spp. are commonly detected and may function either as opportunistic pathogens or as organic matter decomposers [124,125]. For example, De Souza et al. [126] reported Vibrio densities below 20 × 10^2^ CFU mL^−1^ in marine shrimp (*F. brasiliensis*) cultured with molasses as a carbon source, likely due to the ability of sucrose-fermenting Vibrio species to utilize this substrate [127]. In addition, the minimal or zero water exchange typical of BFT systems may favor the accumulation of organic matter, creating conditions conducive to Vibrio persistence [128]. In the present study, *Vibrio* spp. densities did not exceed 10^3^ CFU mL^−1^, a level considered insufficient to induce pathological effects in cultured shrimp [112]. Notably, *Vibrio* spp. were not detected in the chancaca-based treatments, which may be related to differences in the structure and functional activity of the microbial communities established within the biofloc. Biofloc-associated microorganisms may limit the proliferation of opportunistic bacteria through competition for carbon sources, nutrients, and attachment sites, thereby reducing pathogen establishment [129]. Additionally, some biofloc microorganisms have been reported to produce extracellular compounds capable of interfering with bacterial communication mechanisms, such as quorum sensing, potentially modulating Vibrio growth and virulence [130]. However, the present study does not allow causal relationships to be established, and these mechanisms should therefore be interpreted as plausible hypotheses that warrant further experimental validation.

In studies with freshwater fish such as *Ictalurus punctatus* and *Oreochromis niloticus*, as well as with the freshwater shrimp *C. caementarius* cultivated under BFT conditions, phytoplankton communities were found to be dominated by green algae (Chlorophyta), followed by diatoms (Bacillariophyta) and, finally, cyanobacteria (Cyanophyta) [114,131,132], as observed in this study. Regarding zooplankton organisms, the common organisms described in BFT systems were found [60,133,134,135,136], with ciliates being the most abundant, which play an important role in the nutrition of aquatic microorganisms and in controlling bacterial communities [137]. The second most abundant group were rotifers, which are frequently associated with biofloc [26,138,139]. Other organisms present were copepods, which are beneficial due to their content of long-chain polyunsaturated fatty acids (LC-PUFA), minerals, elements, pigments, and free amino acids [140]. Nematodes were the group that presented the lowest abundance, but they are of great importance, and their abundance is determined by the presence of various ciliates that serve as food for them [141,142,143]. The absence of nematodes in the molasses systems may be attributed to the biological substances and probiotics in the biofloc, which have antiparasitic and antihelminthic activities [144]. A higher concentration of microalgae and bacteria was observed in the molasses systems, but a lower concentration of zooplankton compared to the chancaca systems. This could be due to a process of predation or competition between zooplankton organisms and shrimp [89,116,141]. Additionally, it could be associated with a higher amount of TSS and FV in the molasses, which may serve as substrates for bacteria and microalgae [145,146]. In fact, the volume of settleable solids and the total abundance of bacteria were positively correlated with the abundance of flagellated protozoa, indicating an interaction between these microorganism groups [147]. This variation in the composition of organisms, such as heterotrophic bacteria and/or species of zooplankton, could generate variation in the proximate composition of the bioflocs [115,148].

Under BFT conditions, growth performance indices did not clearly reflect the effect of different carbon sources compared to the control. Comparable findings have been reported in other cultured species. For instance, Emerenciano et al. [71], in a study on *F. paulensis* reared under BFT versus a clear-water system, found no improvement in growth performance. The authors suggested that this outcome may be related to the feeding habits of shrimp, as the bioflocs may not have been sufficiently attractive to the species. Similarly, Emerenciano et al. [14] reported that growth performance of the marine shrimp *F. duorarum* did not improve relative to clear-water or water exchange controls. The authors attributed this to several factors, including the species’ lack of domestication, susceptibility to high stocking densities, and lower tolerance to nitrogenous compounds and suspended solids compared with other penaeid shrimp. In the present study, this limitation may also be related to the biological characteristics of *C. caementarius*, which are comparable to those of the prawn *M. rosenbergii*. Both species exhibit behaviors such as cannibalism, territoriality, and the dominance of larger males [149,150,151,152]. In this context, Pérez-Fuentes et al. [153] recommended the application of biofloc technology in *M. rosenbergii* as an alternative strategy, particularly in regions affected by water scarcity where conventional culture systems are not feasible. Similarly, Méndez et al. [152] reported specific growth rate (SGR) values comparable to those obtained in the present study, although associated with lower survival (<38%) under BFT conditions. Survival rates above 60% are generally considered acceptable for freshwater shrimp during juvenile rearing, considering territorial behavior and the well-documented effects of male dominance. According to Valenti et al. [154], survival exceeding 50% between stocking and final harvest is regarded as acceptable in freshwater prawn culture. Regarding feed utilization, the feed conversion ratio (FCR) observed in this study exceeded the range typically reported for shrimp (1.5–2.0) [155,156]. This reduced feed efficiency may be partially attributed to the use of a commercial salmonid diet, selected due to the lack of species-specific formulated feeds for *C. caementarius* at the time of the experiment. The nutritional mismatch of this diet, together with factors such as feed particle loss, limited feed retention within the system, feeding management constraints, and the influence of biofloc dynamics on feeding behavior, may have collectively contributed to the elevated FCR values. Therefore, these results should be interpreted within the biological and methodological constraints of the experimental design rather than as representative of optimized or commercial-scale production conditions. Overall, the present findings indicate that the application of biofloc technology using molasses and chancaca as carbon sources did not result in improvements in growth performance or feed utilization in juvenile *C. caementarius*. Comparisons with marine penaeid shrimp species (e.g., *P. vannamei* and *P. monodon*) should thus be considered strictly contextual, as substantial differences in physiology, feeding behavior, and culture requirements limit direct extrapolation to freshwater crustaceans. Nevertheless, biofloc systems consistently maintained adequate water quality and achieved substantial reductions in water exchange, highlighting their effectiveness as a water-efficient and environmentally sustainable culture strategy. From this perspective, the principal contribution of biofloc technology for *C. caementarius* lies not in maximizing productivity, but in enabling culture under water-limited conditions, which is particularly relevant for endemic freshwater species in arid and semi-arid regions.

It has been described that fish and shrimp in BFT systems can consume flocs, and these may have various effects, such as being a potential source of exogenous enzymes and an enhancer of endogenous enzyme synthesis, which could improve feed digestibility [157,158,159,160,161,162]. Additionally, biofloc has the potential to modify the microbiota in the intestines of the host, which improves absorption capacity, growth performance, and overall health [163,164]. Studies by Cardona et al. [165] on *L. stylirostris* shrimp cultivated in biofloc showed higher enzyme activity levels and greater gene expression for amylase and trypsin, with growth 4.4 times higher than that of shrimp cultivated in clear water. This is in accordance with findings by Anand et al. [29], who measured higher amylase and protease activity in juvenile *P. monodon* cultivated with a biofloc-supplemented diet, resulting in higher growth compared to control systems. It is not possible to distinguish between enzymatic activities synthesized by the shrimp and those produced by microorganisms. However, it is plausible that extracellular enzymes associated with bioflocs are released into the digestive tract upon ingestion by shrimp [159]. Intestinal tissues, which play a critical role in nutrient metabolism, often exhibit histological alterations that serve as reliable biomarkers of toxic conditions [166]. In our study, no positive effect was observed in the aspects mentioned above; however, no negative effects were noted in the process either, indicating no tissue damage or disruption in the secretion of these enzymes [16].

Among the main advantages attributed to biofloc technology (BFT) are its potential to influence physiological responses and general defense mechanisms, as well as to modulate stress tolerance processes and non-specific responses in fish and shrimp cultured in biofloc-based systems [162,167,168]. Heat shock proteins, particularly Hsp70, are among the most extensively studied stress-related biomarkers and are known to increase in response to environmental stressors and pathogen exposure in a wide range of marine invertebrates [169,170,171]. Importantly, Hsp70 should be interpreted as an indicator of general physiological stress and cellular defense, rather than as a direct or specific marker of immune enhancement. In the present study, when Hsp70 levels were examined in relation to bacteriological findings, it was observed that the treatment in which *Vibrio* spp. was detected also exhibited the highest Hsp70 levels; both observations corresponded to the molasses-based BFT treatment at the end of the experimental period. This pattern suggests a trade-off associated with the use of fast-release carbon sources such as molasses, which can promote rapid microbial proliferation and higher microbial loads, potentially increasing metabolic demand and cellular stress in the cultured organisms. This association is strictly correlative and does not imply a causal immunological effect of biofloc or carbon source. The elevated Hsp70 levels likely reflect increased cellular stress or microbial challenge rather than an improvement in immune competence. Previous studies have shown that viral and bacterial challenges can induce changes in Hsp70 expression, including increased transcription of LvHsp70 in *P. vannamei* [172,173]. Similarly, experimental infections of *P. monodon* with *Vibrio harveyi* resulted in significant increases in Hsp70 and Hsp90 expression shortly after exposure, reinforcing the role of heat shock proteins as stress-responsive molecules rather than specific immune effectors [174]. Although biofloc-associated microbial components have been suggested to stimulate non-specific defense mechanisms and improve stress tolerance [175], the present study does not provide sufficient evidence to conclude an immunostimulatory effect. Therefore, changes in Hsp70 expression should be interpreted cautiously and limited to their role as indicators of physiological stress and adaptive cellular responses, rather than as proof of enhanced immunity.

It is important to acknowledge that the control and biofloc treatments differed in water exchange regimes, which represents an inherent methodological contrast between conventional and biofloc-based production systems. This difference was intentional and reflects practical management conditions rather than a controlled manipulation of water exchange. Consequently, the results should be interpreted at the system level, focusing on functional performance, water-use efficiency, and physiological responses, rather than as a direct comparison under identical hydraulic conditions. Traditional shrimp farming can require approximately 64,000 L of water per kilogram of shrimp produced, whereas BFT systems typically operate with substantially lower water inputs, often below 100–200 L per kilogram, depending on management practices [176]. In the present study, the total water exchange volume during the experimental period was 350 L per tank in the biofloc treatments. In contrast, control tanks were subjected to an average of two weekly water exchanges equivalent to 50% of the tank volume, resulting in a cumulative water use of approximately 1900 L per tank over the same period. Consequently, water consumption in the biofloc treatments was 5.4-fold lower than in the control, representing a water savings of 81.6%. These results demonstrate that biofloc-based culture markedly reduces water use compared to conventional clear-water systems, thereby contributing to lower environmental impact and improved resource-use efficiency [177]. Consistent with previous reports, BFT systems are characterized by minimal water exchange and reduced effluent discharge, enabling substantial water savings relative to traditional aquaculture practices [32,178,179,180].

## 5. Conclusions

This study demonstrates that biofloc technology (BFT) can be implemented as an effective water-saving culture strategy for juvenile northern river shrimp (*Cryphiops caementarius*) under the evaluated experimental conditions. The use of molasses and chancaca as fast-release carbon sources supported biofloc development and allowed system operation with minimal water exchange, resulting in an 81.6% reduction in water use compared to a conventional clear-water system.

Importantly, the application of BFT did not lead to significant improvements in growth performance or digestive enzyme activity relative to the control. Growth-related parameters, including final weight, specific growth rate, survival, feed conversion ratio, and productivity, remained comparable among treatments. These results indicate that, for juvenile *C. caementarius*, biofloc systems can maintain biological performance while substantially reducing water consumption, rather than enhancing production metrics. Future studies, addressing the limitations of the present work, should incorporate additional immune-related parameters (e.g., phenoloxidase and lysozyme activities) as well as integrative “omics” approaches such as proteomics and metabolomics. These tools may provide deeper mechanistic insight into host–microbe interactions and physiological stress responses under biofloc conditions, aspects that could not be resolved with the experimental scope and biomarkers employed in this study.

## Figures and Tables

**Table 1:** Water quality parameters of river shrimp (*Cryphiops caementarius*) in biofloc system with different carbon sources during the experimental period.

Water Quality Parameters	Molasses	Chancaca	Control	*p*-Value
Temperature (°C)	21.4 ± 4.0	21.5 ± 3.5	21.7 ± 3.9	0.822
Dissolved Oxygen (mg L ^−1^)	8.62 ± 0.85	8.53 ± 0.73	8.43 ± 0.89	0.323
pH	8.42 ± 0.19	8.37 ± 0.17	8.44 ± 0.19	0.080
TAN (NH_3_ mg L ^−1^)	0.30 ± 0.52	0.12 ± 0.20	0.22 ± 0.44	0.292
Nitrite-N (NO_2_-N mg L ^−1^)	0.75 ± 1.53	0.62 ± 0.95	0.24 ± 0.44	0.161
Nitrate-N (NO_3_-N mg L ^−1^)	71.97 ± 31.38 ^a^	57.87 ± 26.3 ^a^	13.70 ± 8.45 ^b^	0.001
Total Hardness (mg CaCO_3_ L^−1^)	1150.1 ± 304.3 ^a^	1302.6 ± 324.3 ^a^	739.1 ± 124.8 ^b^	0.001
Total alkalinity (mg CaCO_3_ L^−1^)	177.8 ± 32.9 ^b^	185.9 ± 23.9 ^ab^	200.2 ± 20.3 ^a^	0.001
FV (mL L^−1^)	11.71± 6.99	10.86 ± 5.65	Nd	0.784
TSS (mg L^−1^)	132.6 ± 55.2 ^a^	123.3 ± 69.7 ^a^	24.4 ± 15 ^b^	0.001
VSS (mg L^−1^)	61.5 ± 31.2 ^a^	41.8 ± 27.6 ^b^	10.4 ± 8.9 ^c^	0.001

Values are expressed as their mean and standard deviation (n = 3). FV: Floc volume; TSS: total suspended solids. VSS: Volatile Suspended Solids. Nd: not determined. Letters a, b, and c in the same row indicate significant difference with probability factor < 0.05, ANOVA.

**Table 2:** Bacteria composition in biofloc culture with molasses and chancaca as carbon source.

Bacteria Composition	Molasses	Chancaca	*p*-Value
Total bacteria (CFU mL^−1^) × 10^4^	4.97 ± 1.76	1.12 ± 0.31	0.001
*Vibrio* spp. (CFU mL^−1^) × 10^3^	4.28 ± 1.51	0.00 ± 0.00	0.001

Values are expressed as their mean and standard deviation (n = 3). Significant differences among treatments (*p* < 0.05, *t*-test).

**Table 3:** Planktonic abundance in biofloc culture with molasses and chancaca as carbon source.

Microorganism Composition	Molasses	Chancaca	*p*-Value
Phytoplanktonic (cell mL^−1^)			
*Bacillariophyceae*	306.67 ± 45.23	139.67 ± 12.89	0.001
*Chlorophyceae*	334.67 ± 29.50	233.67 ± 55.72	0.001
*Cyanophyceae*	7.33 ± 5.51	1.67 ± 0.58	0.001
Zooplanktonic			
Rotifer (ind. mL^−1^)	3.67 ± 1.15	7.33 ± 5.51	0.061
*Philodina* sp.	1.33 ± 0.58	2.67 ± 0.58	0.254
*Lecane* sp.	1.67 ± 0.58	3.00 ± 1.73	0.290
*Cephalodella* sp.	1.00 ± 0.00	1.67 ± 0.58	0.200
Cladocera (ind. mL^−1^)	0.33 ± 0.57	0.00 ± 0.00	0.001
*Chydorus* sp.	0.33 ± 0.57	0.00 ± 0.00	0.001
Nematode(ind. mL^−1^)	0.00 ± 0.00	0.67 ± 0.57	0.001
*Monhystera* sp.	0.00 ± 0.00	0.67 ± 0.57	0.001
Copepod (ind. mL^−1^)	0.33 ± 0.57	0.67 ± 0.57	0.001
*Cyclops* sp.	0.33 ± 0.57	0.67 ± 0.57	0.001
Amoeba (ind. mL^−1^)	0.67 ± 0.57	0.67 ± 0.57	1.000
*Amoeba* sp.	0.67 ± 0.57	0.67 ± 0.57	1.000
Ciliates (ind. mL^−1^)	32.33 ± 7.51	37.00 ± 5.19	0.333
*Paramecium* sp.	6.00 ± 1.00	4.00 ± 1.00	0.275
*Coleps* sp.	10.33 ± 1.52	18.00 ± 2.00	0.010
*Vorticella* sp.	5.67 ± 2.09	7.67 ± 4.04	0.343
*Halteria* sp.	3.67 ± 3.79	1.67 ± 0.58	0.133
*Euplotes* sp.	6.67 ± 2.52	5.67 ± 1.53	0.628

Values are expressed as their mean and standard deviation (n = 3). Planktonic organisms were identified to the lowest feasible taxonomic level based on morphological criteria. Statistical differences were assessed using the independent samples *t*-test. Significant differences among treatments (*p* < 0.05). Significant differences should be interpreted with caution due to low absolute abundances of some taxa.

**Table 4:** Growth performance of river shrimp (*Cryphiops caementarius*) in biofloc system with different carbon sources during the experimental period.

Parameters	Molasses	Chancaca	Control	*p*-Value
Initial body weight (g)	0.83 ± 0.56	0.88 ± 0.71	0.85 ± 0.68	0.711
Final body weight (g)	2.10 ± 2.50	2.39 ± 3.68	2.37 ± 3.64	0.764
Survival (%)	68.60 ± 4.25	60.10 ± 5.19	66.10 ± 11.10	0.422
Feed conversion ratio (FCR)	6.27 ± 0.37	6.86 ± 0.53	5.45 ± 0.732	0.400
Specific growth rate (% day^−1^)	0.58 ± 0.12	0.63 ± 0.04	0.65 ± 0.04	0.633
Productivity (g m^−2^)	104.52 ± 16.79	104.22 ± 15.65	113.09 ± 20.21	0.485

Values are expressed as their mean and standard deviation (n = 3). Significant differences among treatments (*p* < 0.05, Kruskal–Wallis test).

**Table 5:** Digestive enzyme activity of river shrimp (*Cryphiops caementarius*) in a biofloc system with different carbon sources.

Parameter	Digestive Enzyme Activity			*p*-Value
	Molasses	Chancaca	Control	
Amylase (IU mL)	6.73 ± 1.64	5.88 ± 1.82	7.55 ± 2.64	0.200
Trypsin (IU mL)	1.89 ± 1.07	2.80 ± 2.32	2.03 ± 0.94	0.851

Values are expressed as their mean and standard deviation (n = 12). Significant differences among treatments (*p* < 0.05, Kruskal–Wallis test).

## Data Availability

Data are contained within the article.
